# Characterization and impact of non‐canonical WNT signaling on outcomes of urothelial carcinoma

**DOI:** 10.1002/cam4.7148

**Published:** 2024-04-01

**Authors:** Margaret Meagher, Harris Krause, Andrew Elliott, Alex Farrell, Emmanuel S. Antonarakis, Bruno Bastos, Elisabeth I. Heath, Christina Jamieson, Tyler F. Stewart, Aditya Bagrodia, Chadi Nabhan, Matt Oberley, Rana R. McKay, Amirali Salmasi

**Affiliations:** ^1^ Department of Urology UC San Diego School of Medicine La Jolla California USA; ^2^ Caris Life Sciences Phoenix Arizona USA; ^3^ Division of Hematology, Oncology, and Transplantation University of Minnesota Minnesota USA; ^4^ Miami Cancer Institute Miami Florida USA; ^5^ Karmanos Cancer Institute, Department of Oncology Wayne State University School of Medicine Detroit Michigan USA; ^6^ Department of Medicine UC San Diego School of Medicine La Jolla California USA; ^7^ Barbara Ann Karmanos Cancer Institute Detroit USA

**Keywords:** FZD2, Non‐canonical, ROR, Urothelial carcinoma, WNT signaling, WNT5A

## Abstract

**Background:**

Non‐canonical WNT family (WNT5A pathway) signaling via *WNT5A* through *ROR1* and its partner, *ROR2*, or Frizzled2 (*FZD2*) is linked to processes driving tumorigenesis and therapy resistance. We utilized a large dataset of urothelial carcinoma (UC) tumors to characterize non‐canonical WNT signaling through *WNT5A*, *ROR1*, *ROR2*, or *FZD2* expression.

**Methods:**

NextGen Sequencing of DNA (592 genes or WES)/RNA (WTS) was performed for 4125 UC tumors submitted to Caris Life Sciences. High and low expression of *WNT5A*, *ROR1*, *ROR2*, and *FZD2* was defined as ≥ top and <bottom quartile of transcripts per million (TPM), respectively. Gene expression profiles were analyzed for a transcriptional signature predictive of response to immunotherapy. Mann–Whitney *U* and X2/Fisher Exact tests were applied where appropriate, with *p*‐values adjusted for multiple comparisons (*p* < 0.05). Real‐world overall survival (OS) was obtained from insurance claims data.

**Results:**

WNT5A pathway gene expression varied significantly between primary versus metastatic sites: *WNT5A* (25.2 vs. 16.8 TPM), *FZD2* (3.2 vs. 4.05), *ROR1* (1.7 vs. 2.1), and *ROR2* (2.4 vs. 2.6) *p* < 0.05 for all. Comparison of high‐ and low‐expression subgroups revealed variation in the prevalence of *TP53*, *FGFR3*, and *RB1* pathogenic mutations, as well as increasing T cell‐inflamed scores as expression of the target gene increased. High gene expression for *ROR2* (HR 1.31, 95% CI 1.15–1.50, *p* < 0.001) and *FZD2* (HR 1.16, 95% CI 1.02–1.32, *p* = 0.024) was associated with worse OS.

**Conclusion:**

Distinct genomic and immune landscapes for the four investigated WNT5A pathway components were observed in patients with UC. External validation studies are needed.

## 1 INTRODUCTION

The WNT pathway can be activated by secreted glycoproteins that play critical roles both in embryonic development and in normal physiologic functions.^1^ Abnormal WNT signal transduction has been associated with disease states such as cancer and autoimmune conditions.^2^, ^3^ The WNT pathway is complex and can be divided into either canonical (β‐catenin dependent) or non‐canonical (β‐catenin independent) signaling pathways involving 19 WNT ligands, 10 Frizzled (FZD) receptors, and various non‐FZD receptors.^3^, ^4^, ^5^ The canonical signaling pathway is known to be β‐catenin‐dependent and modulate the tumor immune microenvironment via Wnt1, Wnt3a, or Wnt8 proteins.^3^, ^4^ There are also several distinct non‐canonical WNT pathways, which generally stimulate β‐catenin‐independent pathways through Wnt5A, Wnt5B, and Wnt11 proteins. These pathways generally activate Planar Cell Polarity (PCP) signals, receptor tyrosine kinase (RTK) family, or Ca + 2 signaling cascades via FZD and other co‐receptors, such as Receptor tyrosine kinase‐like Orphan Receptor (ROR)1 and ROR2.^3^, ^5^

WNT signaling via the ROR family of proteins has gained increasing attention in cancer research due to overexpression in tumor cells.^2^ In adult tissue, the RORs are largely absent, which makes them ideal for targeted therapies.^2^ While the impact of *WNT5A* on cancer pathogenesis is context dependent, increasing studies suggest it has a negative effect on tumorigenesis and the corresponding tumor microenvironment (TME).^5^, ^6^ It has been shown that upregulation of WNT signaling via *WNT5A* through *ROR1* and *ROR2* can induce metabolic reprogramming and immune dysregulation in TME, which promotes cancer progression in various malignancies.^2^, ^5^, ^7^, ^8^ Similarly, *WNT5A* signaling via the *FZD2* receptor has been associated with processes such as cell proliferation, dysregulation of tumor microenvironment, cell migration, and therapy resistance that contributes to tumorigenesis.^9^, ^10^

The role of the WNT5A pathway (*WNT5A*, *ROR1*, *ROR2*, and *FZD2*) in the pathogenesis and progression of urothelial carcinoma (UC) has not been fully elucidated. In vivo studies have demonstrated the feasibility of WNT signaling pathway inhibitors, and several clinical trials investigating are actively recruiting patients with a variety of malignancies.^3^, ^11^, ^12^ This highlights the importance of understanding the genomic and immunologic landscape associated with different pathway components to inform treatment strategies.^3^ We utilized a large dataset of molecular profiled UC tumors to investigate the significance of *WNT5A*, *ROR1*, *ROR2*, or *FZD2* expression. We characterized molecular alterations associated with the expression of WNT5A pathway components investigated the association of expression of WNT5A pathway components with the immunologic landscape and inferred real‐world clinical outcomes.

## 2 PATIENTS AND METHODS

### 2.1 Study cohort

We retrospectively analyzed a large dataset of molecularly profiled UC (*N* = 4125), breast (*N* = 11,246), colorectal (*N* = 15,425), head and neck (*N* = 3317), melanoma (*N* = 3424), non‐small lung cancer (*N* = 21,603), pancreatic (*N* = 5488), and prostate (*N* = 5500) tumors that were submitted to a CLIA certified genomics laboratory (Caris Life Sciences, Phoenix, AZ) to investigate the significance of *WNT5A*, *ROR1*, *ROR2* or *FZD2* transcriptional expression. The present study was conducted in accordance with the guidelines of the Declaration of Helsinki, the Belmont Report, and US Common Rule. In compliance with policy 45 CFR 46.101(b), this study was conducted using retrospective, de‐identified clinical data, and patient consent was not required.

### 2.2 Defining tumor site

A primary (local) tumor was defined as one with biopsies collected from the annotated primary site. A metastatic tumor was defined as any non‐primary tumor. Lower and upper urothelial tract (LT and UT) was defined by the annotated primary site and specimen (biopsy) site. UT corresponds to tumors arising in the kidney and ureter while LT refers to tumors arising in the urinary bladder and/or urethra. Of tumors that had a UT/LT annotation, 795 tumors were UT, and 3204 tumors were LT. Of tumors that had a primary or metastatic annotation, 2756 tumors were from primary sites, and 1361 tumors were from metastatic sites.

### 2.3 Immunohistochemistry (IHC)

IHC was performed on full formalin‐fixed paraffin‐embedded (FFPE) sections of glass slides. Slides were stained using automated staining techniques, per the manufacturer's instructions, and were optimized and validated per CLIA/CAP and ISO requirements. Staining was scored for intensity (0 = no staining; 1+ = weak staining; 2+ = moderate staining; 3+ = strong staining) and staining percentage (0%–100%). PD‐L1 (SP142) positive (+) staining was defined as ≥2+ and ≥5%.

### 2.4 DNA next‐generation sequencing (NGS)

A targeted 592‐gene panel or whole exome sequencing (WES) was performed using genomic DNA isolated from FFPE tumor samples. The 592‐gene panel was sequenced using the NextSeq platform (Illumina, Inc., San Diego, CA). A custom‐designed SureSelect XT assay was used to enrich 592 whole‐gene targets (Agilent Technologies, Santa Clara, CA). WES was performed using the Illumina NovaSeq 6000 sequencer (Illumina, Inc). A hybrid pull‐down panel of baits designed to enrich for 700 clinically relevant genes at high coverage and high read‐depth was used, along with another panel designed to enrich for additional >20,000 genes at a lower depth. In this study, 592‐gene and WES assays were cross‐validated and showed highly concordant results. Matched normal tissue was not sequenced.

### 2.5 Identification of genetic variants

Genetic variants identified were interpreted by board‐certified molecular geneticists and categorized as “pathogenic,” “likely pathogenic,” “variant of unknown significance,” “likely benign,” or “benign,” according to the American College of Medical Genetics and Genomics (ACMG) standards. When assessing mutation frequencies of individual genes, “pathogenic” and “likely pathogenic” were counted as mutations while “benign,” “likely benign” variants, and “variants of unknown significance” were excluded.

### 2.6 Whole transcriptome sequencing

All samples included in this study underwent transcriptomic sequencing. FFPE specimens underwent pathology review to diagnose percent tumor content and tumor size; a minimum of 10% of tumor content in the area for microdissection was required to enable enrichment and extraction of tumor‐specific RNA. Qiagen RNA FFPE tissue extraction kit was used for extraction, and the RNA quality and quantity were determined using the Agilent TapeStation. Biotinylated RNA baits were hybridized to the synthesized and purified cDNA targets and the bait‐target complexes were amplified in a post‐capture PCR reaction. The resultant libraries were quantified, normalized and the pooled libraries were denatured, diluted, and sequenced; the reference genome used was GRCh37/hg19 and analytical validation of this test demonstrated ≥97% positive percent agreement (PPA), ≥99% negative percent agreement (NPA), and ≥99% Overall Percent Agreement (OPA) with a validated comparator method.

For transcript counting, transcripts per million (TPM) molecules were generated using the Salmon expression pipeline. High and low expression of *WNT5A*, *ROR1*, *ROR2*, and *FZD2* was defined as ≥ top and <bottom quartile of transcripts per million, respectively.

### 2.7 Immune signatures

Immune cell fractions were estimated by RNA deconvolution using quanTIseq.^13^ The T cell‐inflamed score was calculated using 160‐gene expression signatures as described by Bao et al (inflamed ≥80, intermediate >−80 and <80, not inflamed −≤80), which was previously shown to correlate with increased response to immunotherapy.^14^

### 2.8 Clinical outcomes

Real‐world clinical outcomes were inferred using insurance claims data. Survival was calculated from the date of tissue collection or first treatment with immune checkpoint inhibitors (avelumab, atezolizumab, nivolumab, or pembrolizumab) until the date of last contact (100 days since the last insurance claim). Date of tissue collection was used as it provides a standardized and consistent starting point for all individuals in the study and often represents a clinically relevant event. In a subgroup analysis comparing the last contact inferred from insurance claims with natural death from the national death index, the concordance was >90%. High and low expression subgroups were defined by the top and bottom quartile of transcripts per million (TPM), respectively. The Kaplan–Meier method was used to estimate survival functions, with survival distributions compared using the log‐rank test.

### 2.9 Data analysis

Fisher exact test (R V.3.6.1), χ^2^, and Mann–Whitney U (scipy V.1.9.3) test were used for statistical analysis, and all *p*‐values were adjusted for multiple comparisons with *p* < 0.05 considered significant.

## 3 RESULTS

### 3.1 Study population

**FIGURE 1:**
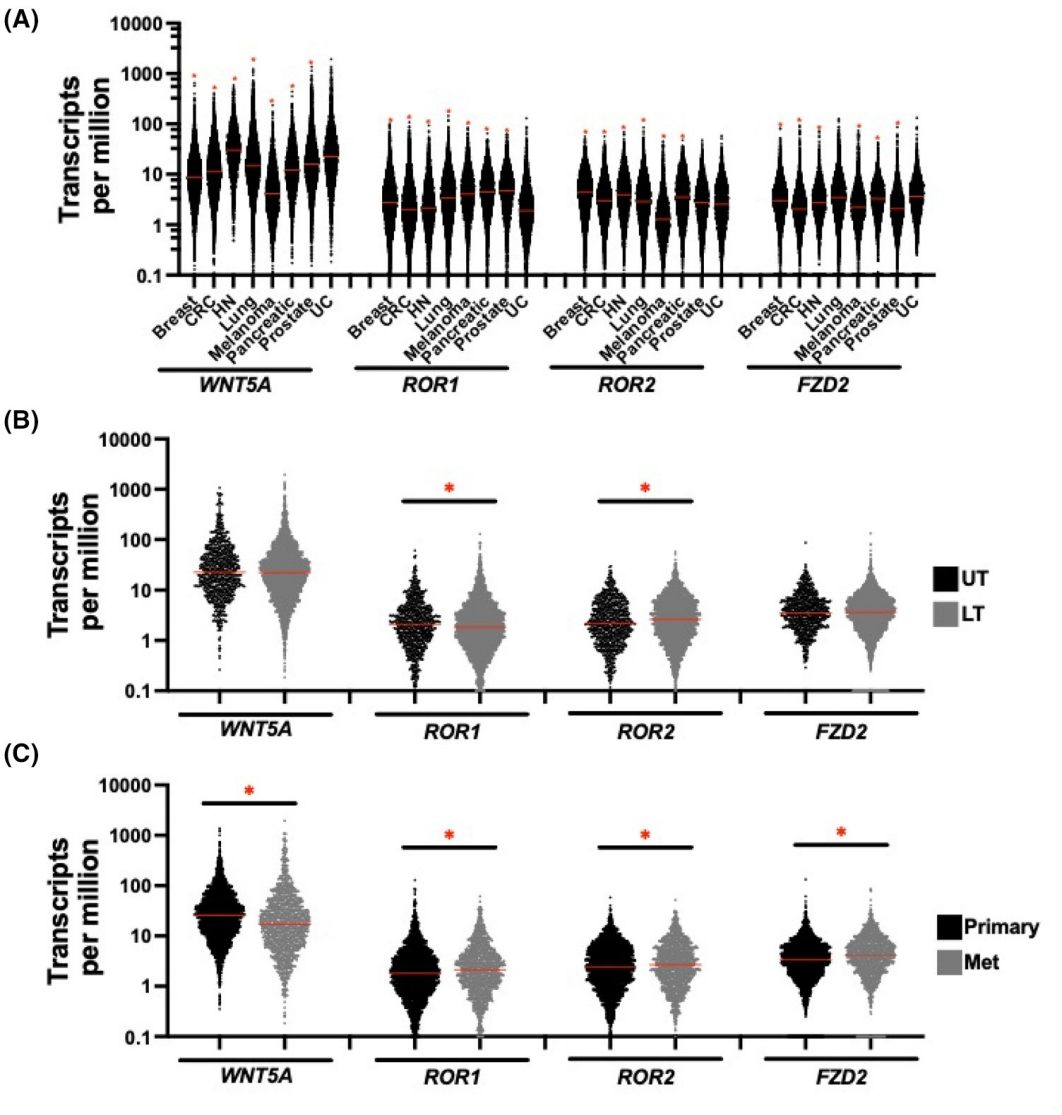
Expression of key WNT5A pathway genes (transcripts per million) (A) across several solid tumor types, (B) upper versus lower UC, and (C) primary versus metastatic UC. For (A), red asterisk indicates that expression in that tumor type is significantly different than expression in UC (*p* < 0.05). For (B, C) bar and red asterisks indicate statistically significant differences (*p* < 0.05). The red line denotes the median.

**FIGURE 2:**
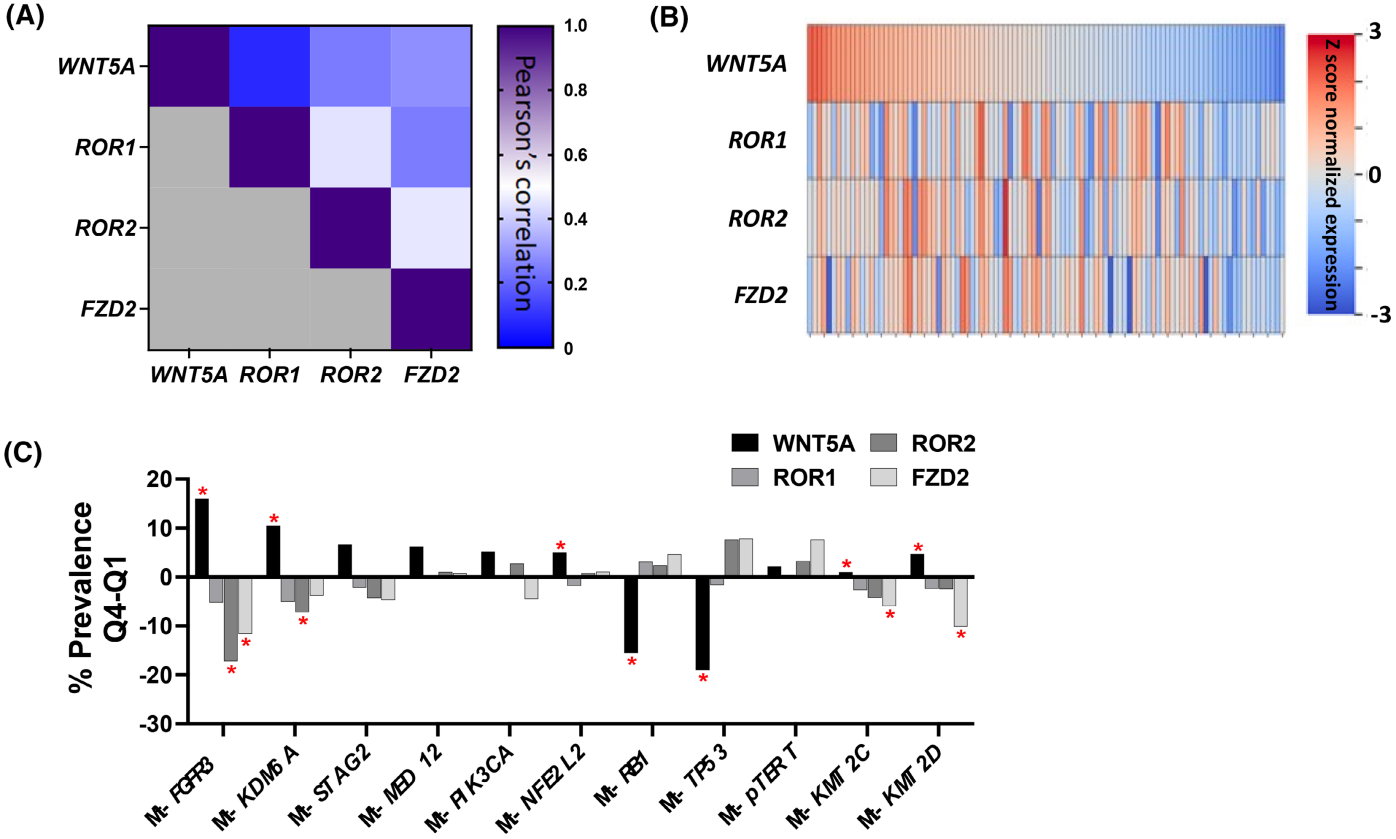
(A) Pearson's correlation between the log10 (gene expression+0.1) of WNT5A pathway genes of interest. (B) Z score normalized expression of WNT5A pathway genes of interest across 100 tumors. (C) Difference in prevalence of selected mutations between high or low expression of WNT5A pathway genes (genes shown have >5% difference in prevalence between Q4 and Q1 of indicated gene, red asterisk indicates *p* < 0.05).

As there is no consensus on what constitutes normal, low, or high levels of WNT5A genes, we have provided this figure to depict a comparison across various tumor types (Figure 1A). WNT5A pathway gene expression varied slightly across solid tumors. The upper tract tumors had significantly higher *ROR1* and *ROR2* expression compared with lower tract UC *ROR1* (2.0 vs. 1.7 TPM, *p* = 0.047) and *ROR2* (2.1 vs. 2.5 TPM, *p* < 0.01) (Figure 1B). *WNT5A* pathway gene expression varied significantly between primary and metastatic sites. *WNT5A* had higher expression in the primary site (25.2 vs. 16.8 TPM) and *FZD2* (3.2 vs. 4.05 TPM), *ROR1* (1.7 vs. 2.1 TPM), and *ROR2* (2.4 vs. 2.6 TPM) had higher expression in the metastatic site (*p* < 0.05 for all) (Figure 1C) in addition to across metastatic sites (Figure S1). There were no significant differences between the top (high) and bottom (low) quartile with respect to median age or sex across all gene subgroups (Table 1). Additionally, a high level of correlation (Pearson) between *ROR1*/*ROR2* (*R* = 0.44) and *ROR2*/*FZD2* (*R* = 0.45) expression was identified as compared to the other genes of interest (Figure 2A,B). This correlation was maintained regardless of the primary/metastatic status of the tumor (data not shown).

**TABLE 1:** Median expression (transcripts per million) of WNT5A pathway genes of interest based on clinical demographics, including age, gender, upper versus lower tract UC, and primary versus metastatic disease.

	*WNT5A* Q1	*WNT5A* Q2	*WNT5A* Q3	*WNT5A* Q4	Statistic	*q*‐Value
Count (*N*)	1032	1031	1031	1031		
Median age [range] (*N*)	72 [26 to >89] (1032)	72 [28 to >89] (1031)	72 [18 to >89] (1031)	73 [24 to >89] (1031)	Kruskal‐Wallis	0.37
Male	73.4% (757/1032)	73.2% (755/1031)	72.2% (744/1031)	69.4% (716/1031)	Chi‐square	0.28
% Primary	55.0% (565/1028)	67.1% (691/1030)	73.4% (755/1029)	72.3% (745/1030)	Chi‐square	<0.001
Upper vs. lower tract (% upper tract)	18.7% (186/994)	25.6% (205/1005)	18.5% (186/1003)	21.9% (218/997)	Chi‐square	0.81

	*ROR1* Q1	*ROR1* Q2	*ROR1* Q3	*ROR1* Q4	Statistic	*q*‐Value
Count (*N*)	1032	1031	1031	1031		
Median age [range] (*N*)	72 [26 to >89] (1032)	72 [28 to >89] (1031)	72 [18 to >89] (1031)	73 [24 to >89] (1031)	Kruskal‐Wallis	0.37
Male	73.4% (757/1032)	73.2% (755/1031)	72.2% (744/1031)	69.4% (716/1031)	Chi‐square	0.28
% Primary	71.7% (737/1028)	67.2% (692/1030)	66.1% (681/1030)	62.8% (646/1029)	Chi‐square	0.001
Upper vs. lower tract (% upper tract)	17.2% (171/996)	19.6% (197/1007)	21.3% (213/1001)	21.5% (214/995)	Chi‐square	0.23

	*ROR2* Q1	*ROR2* Q2	*ROR2* Q3	*ROR2* Q4	Statistic	*q*‐Value
Count (*N*)	1032	1031	1031	1031		
Median age [range] (*N*)	72 [18 to >89] (1032)	73 [26 to >89] (1031)	72 [27 to >89] (1031)	72 [34 to >89] (1031)	Kruskal‐Wallis	0.29
Male	70.7% (730/1032)	72.2% (744/1031)	71.0% (732/1031)	74.3% (766/1031)	Chi‐square	0.29
% Primary	69.4% (714/1029)	66.9% (688/1029)	66.4% (685/1031)	65.1% 669/1028	Chi‐square	0.84
Upper vs. lower tract (% upper tract)	22.6% (227/1004)	21.6% (216/1000)	18.0% (178/991)	17.3% (174/1004)	Chi‐square	0.02

	FZD2 Q1	FZD2 Q2	FZD2 Q3	FZD2 Q4	Statistic	*q*‐Value
Count (*N*)	1032	1031	1031	1031		
Median age [range] (*N*)	72 [18 to >89] (1032)	72 [24 to >89] (1031)	72 [27 to >89] (1031)	72 [32 to >89] (1031)	Kruskal‐Wallis	0.90
Male	72.8% (751/1032)	71.0% (732/1031)	71.1% (733/1031)	73.3% (756/1031)	Chi‐square	0.68
% Primary	75.0% (757/1009)	68.9% (709/1029)	65.9% (679/1030)	59.4% (611/1029)	Chi‐square	<0.001
Upper vs. lower tract (% upper tract)	19.5% (195/1001)	21.0% (210/1000)	19.6% (195/994)	19.4% (195/1004)	Chi‐square	1.00

### 3.2 Genomic landscape of WNT5A pathway genes

The genomic landscapes of high‐ (Q4) and low (Q1)‐expressing WNT5A pathway genes were investigated. We focused on mutations that had at least a 5% difference in prevalence between low‐ and high‐expressing tumors (Figure 2C). Between low‐ and high‐expressing *WNT5A* tumors, *TP53* (67% vs. 48%, *p* < 0.001) and *RB1* mutations (30% vs. 15%, *p* < 0.001) had a higher prevalence in low‐expressing tumors whereas *FGFR3* mutations had an increased prevalence in high‐expressing tumors (9% vs. 25%, *p* < 0.001). When segmenting based on *ROR1* expression, there were no significant differences in the prevalence of *TP53* (*p* = 1.00), *FGFR3* (*p* = 0.48), or *RB1* (*p* = 1.00) alterations between low and high expression subgroups. The prevalence of *FGFR3* mutations was significantly higher in low versus high *ROR2* expressors (25% vs. 7%, *p* < 0.001), but no significant differences in the prevalence of *TP53* (52% vs. 60%, *p* = 0.10) nor *RB1* (22% vs. 24%, *p* = 1.00) alterations were observed. Similarly, between the *FZD2* low and high subgroups, there was a significantly higher prevalence of *FGFR3* mutation in the low group (21% vs. 9%, *p* < 0.001), but no significant differences in the prevalence of *TP53* (56% vs. 64%, *p* = 0.10), nor *RB1* (22% vs. 26%, *p* = 1.00) mutations (Figure 2C). When analyzing just UT or LT UC, the genomic landscapes of high (Q4) and low (Q1) expressing WNT5A pathway genes showed similar trends (Figure S2).

### 3.3 Tumor immune microenvironment

**FIGURE 3:**
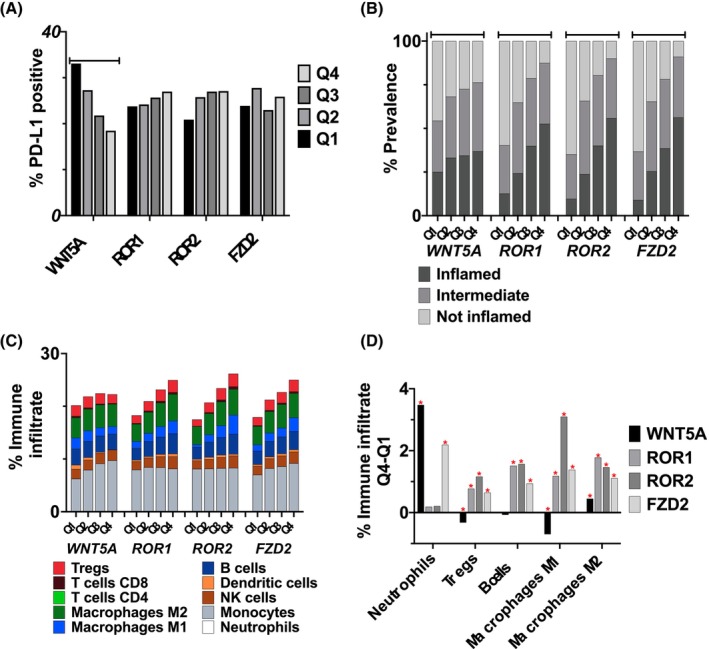
(A) Prevalence of PD‐L1+ IHC staining across expression quartiles of investigated *Wnt*5A genes (asterisk indicates *p* < 0.05) (B) % of tumors that are considered inflamed, intermediate, or not inflamed based on their t‐cell inflamed score (asterisk indicates *p* < 005). (C) % tumor immune infiltrate across expression quartiles of investigated WNT5A pathway genes. (D) Difference in expression of immune infiltrate between Q4 and Q1 of the indicated WNT5A pathway gene (asterisk indicates *p* < 0.05).

**FIGURE 4:**
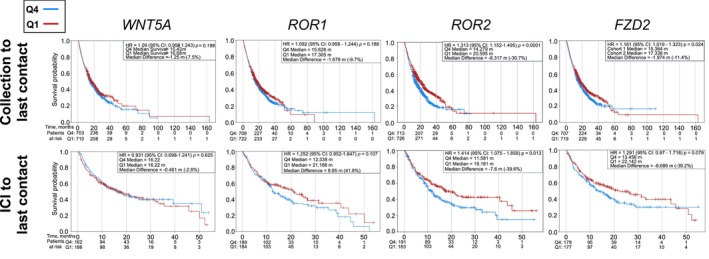
Overall survival and time from immune checkpoint inhibitor (ICI) to last contact for the indicated genes.

Low *WNT5A* expressors had a significantly higher rate of PD‐L1+ tumors as compared to high expressors (33.1% vs. 18.5% *p* < 0.001). When stratifying by expression quartiles for the other WNT family genes no imbalance in the prevalence of PD‐L1+ tumors was observed (Figure 3A). This pattern was also observed regardless of whether the tumor was classified as primary or metastatic except in Primary tumors when segmenting by ROR2 where high ROR2 expressors had a significantly higher prevalence of PDL1+ tumors as compared to low expressors (29.6% vs. 19.3%, *p* = 0.002). Across all investigated genes, tumors with high expression were significantly more likely to be considered inflamed by the T cell‐inflamed score (Figure 4B). We investigated the percent immune infiltrate across high and low expression of our target genes using immune deconvolution (Figure 4C,D). Investigating the difference in lymphoid‐derived immune cell infiltrate, there was a significantly higher fraction of NK, B, Treg, and CD8+ T cell infiltrate amongst high expressors of *ROR1*, *ROR2*, and *WNT5A*. Next, we investigated myeloid‐derived cells. For *WNT5A* and *FZD2* genes, there was significantly more neutrophil infiltrate in high‐ as compared to low‐expressing tumors. For *ROR1*, *ROR2*, and *FZD2* genes, there was significantly more M1 and M2 macrophage immune infiltrate in high as compared to low expressors.

### 3.4 Treatment and outcome analysis

*WNT5A* and *ROR1* gene expression was not significantly associated with survival outcomes. High gene expression of *ROR2* was associated with worsened overall survival (HR = 1.31, *p* < 0.001) and worse overall survival in the setting of immunotherapy (HR = 1.41, *p* = 0.013). High gene expression of *FZD2* was also associated with worse overall survival (HR = 1.16, *p* = 0.024), but not worse overall survival in the setting of immunotherapy (HR = 1.291, *p* = 0.079) (Figure 4), the lack of significance in the setting of immunotherapy is likely due to a lower *N* (*N* = 1426 vs. *N* = 356). These results were similar when separating primary and metastatic tumors (Figures S1 & S2).

## 4 DISCUSSION

In our study, the expression of *ROR1*/ *ROR2* and *ROR2*/*FZD2* were highly correlated. ROR1, virtually absent in normal adult tissues, exhibits activity in various malignancies and has been linked to tumor cell growth.^7^ Due to its selective expression, *ROR1* is an enticing pathway component as targeting avoids the deleterious effects of non‐selective agents. Importantly, as transmembrane cell surface proteins, *ROR1/2* and *FZD2* represent targets for antibody‐drug conjugates. Slight differences in the expression of WNT pathway components were observed between primary versus metastatic tumors and between upper versus lower tract tumors. Although these results were significant the effect size was small and the sample size was relatively large so it is unclear how biologically relevant these differences are.

Tumors with high expression of *WNT5A* had an increased prevalence of *FGFR3* mutations. In comparison, tumors with low expression of ROR2 or FZD2 also had an increased prevalence of *FGFR3* mutations. This is intriguing because *FGFR3* is a targetable mutation with selective inhibitors in the clinic.^15^, ^16^, ^17^ Previous research has estimated that about 50% of bladder cancers have somatic alterations in FGFR3 coding sequences.^18^ Presence of FGFR3 has been noted to be mutually exclusive with other mutations and thus may represent a distinct therapeutic target for oncogenesis prevention.^15^ In a xenograft mice model, Jing et al demonstrated that FGFR3 inhibitors lead to an upregulation of PD‐L1 expression.^17^ This inverse relationship was also noted in our study whereby high WNT5A expressors, which were correlated with increased FGFR3 mutation prevalence, had a significantly lower PD‐L1+ positivity rate. The combination of *FGFR3* inhibitors and therapies targeting *WNT5A* should be further investigated. Lower expression of *WNT5A* was associated with increased *RB1* and *TP53* mutations. Past studies suggest that *TP53* and *RB1* alterations have prognostic value and can predict response to systemic immunotherapy.^18^, ^19^

In the metastatic setting, only about 30% of UC patients will respond to the current immune checkpoint inhibitor paradigm.^20^ Sweis et al posited that β‐catenin pathways were activated in non‐T cell‐inflamed UC phenotypes, whereby immune cells are excluded from the tumor microenvironment, which carries inherent resistance to current immunotherapeutic agents.^21^ Luke et al validated these findings suggesting that upregulation of β‐catenin pathway correlates with an immunosuppressive tumor microenvironment.^22^ In our study, we found that high expression of *WNT5A* was associated with a higher prevalence of T cell‐inflamed tumors, which has been shown to strongly predict response to immunotherapy.^22^ Conversely, low expression of *WNT5A* was also associated with high PD‐L1 positivity rates and mutations (*TP53* and *RB1*) associated with response to ICI.^18^, ^19^ Interestingly, high expression of *ROR1*, *ROR2*, and *FZD2* were associated with a higher proportion of T cell‐inflamed tumor. However, high expression of *ROR1*, *ROR2*, and *FZD2* were also associated with increased myeloid infiltration which has been demonstrated to be immunosuppressive.^23^ Our work revealed mixed signals regarding the responsiveness of tumors highly expressing the WNT pathway to immunotherapy. Further follow‐up investigation is needed in this area.

*WNT5A* expression was not associated with improved overall survival. In contrast, previous research showed that increased *WNT5A* expression is associated with worsened prognostic indicators in UC.^24^, ^25^ Bayat et al demonstrated that an anti‐ROR1 monoclonal antibody (F1‐B10) induced apoptosis in two human bladder cell lines, and thus proposed that ROR1 may play a role in bladder cancer cell survival.^9^ Our data did not support this hypothesis, showing no difference in overall survival based on *ROR1* expression. Yeh et al investigated the expression of *ROR2* expression through transcriptomic profiling of a published dataset (GSE31684) and 50 frozen bladder UCs.^26^ They showed that high *ROR2* expression was associated with aggressive pathological characteristics in UC and independently predicted worse prognoses. Our data agrees with the literature, high *ROR2* expression was associated with worse outcomes. Future work should focus on the feasibility of targeting *ROR2*. Finally, we showed that *FZD2* expression was associated with worse overall survival. This is an exciting observation as *FZD2*‐targeted therapies are beginning to enter the clinic.^12^

Our study is limited by the inherent constraints of a retrospective study. Importantly, this database does not include tumor grade and stage, presence of variant histology, or patient comorbidities, all of which may affect survival. Data regarding chemotherapy and the timing of immunotherapy are also missing. Insurance claims data was used to infer overall survival (biopsy collection to last contact) and the effect of patient comorbidities and other competing risks were unable to be taken into account. We did not have single‐cell sequencing data to characterize the exhaustion of CD8 cells. Furthermore, the single immune signature used in this study may not capture all aspects of T‐cell modulation. Despite these limitations, this is the first study to evaluate the role of a non‐canonical WNT pathway in large dataset of molecular profiling of UC tumors.

## 5 CONCLUSION

This study identified clinically relevant mutations in *FGFR3*, *TP53*, and *RB1* that are associated with expression of WNT5A pathway genes. High expression of *ROR2* and *FZD2* were associated with decreased overall survival. Distinct genomic and immune landscapes for the four investigated WNT5A pathway genes were observed and should be leveraged to identify combination therapies that complement the current pipeline of WNT pathway‐targeting drugs. External validation studies are needed.

## AUTHOR CONTRIBUTIONS

**Margaret Meagher:** Conceptualization (equal); investigation (equal). **Harris Krause:** Conceptualization (equal); data curation (equal); formal analysis (equal). **Andrew Elliott:** Conceptualization (equal); data curation (equal); formal analysis (equal). **Alex Farrell:** Conceptualization (equal); data curation (equal); formal analysis (equal). **Emmanuel S. Antonarakis:** Conceptualization (equal). **Bruno Bastos:** Conceptualization (equal). **Elisabeth I. Heath:** Conceptualization (equal). **Christina Jamieson:** Conceptualization (equal). **Tyler F. Stewart:** Conceptualization (equal). **Aditya Bagrodia:** Conceptualization (equal). **Chadi Nabhan:** Conceptualization (equal); data curation (equal); formal analysis (equal). **Matt Oberley:** Conceptualization (equal); data curation (equal); formal analysis (equal). **Rana R. McKay:** Conceptualization (equal). **Amirali Salmasi:** Conceptualization (lead); methodology (equal); project administration (equal).

## FUNDING INFORMATION

None.

## CONFLICT OF INTEREST STATEMENT

Harris Krause, Andrew Elliott, Alex Farrell, Chadi Nabhan, and Matt Oberley are affiliated with Caris Life Sciences.

## ETHICS STATEMENT

Our commitment includes transparent data availability, acknowledgment of funding sources, disclosure of any potential conflicts of interest, adherence to rigorous ethical protocols, and meticulous permissions for material reproduction. These principles guide our unwavering dedication to integrity in all our research pursuits.

## PRECIS

This study unveils the distinctive features and consequential effects of non‐canonical WNT signaling in Urothelial Carcinoma. The findings illuminate its characterization and underscore its pivotal role in shaping disease outcomes, providing critical insights for targeted therapeutic interventions.

## Supporting information


Figure S1.
Figure S2.
Figure S3.

## Data Availability

The data used in this study is not publicly available but can be made available upon reasonable request of the lead author (AS).
