# Supplementary material for: Characterization and impact of non‐canonical WNT signaling on outcomes of urothelial carcinoma
Source: Cancer Med. 2024 Apr 1;13(7):e7148. doi: 10.1002/cam4.7148 (PMC10983807; doi:10.1002/cam4.7148)
Supplement: Supplementary file 1 — Figure S1. Figure S2. Figure S3. [file CAM4-13-e7148-s001.docx]

**Supplement 1**: Expression of genes (TPM) of interest across metastatic sites, red asterisk indicates *q* < 0.05.

**Supplement 2**: Difference in prevalence of selected mutations between high or low expression of *Wnt* pathway genes in Upper tract **(A)** and Lower Tract **(B)** UC (genes shown were showcased in figure 2, red asterisk indicates *q* < 0.05).

**Supplement 3:** Genomic landscape of Urothelial Carcinoma, segmented by expression of the indicated gene.
